# Methods and Best Practice to Intercompare Dissolved Oxygen Sensors and Fluorometers/Turbidimeters for Oceanographic Applications

**DOI:** 10.3390/s16050702

**Published:** 2016-05-17

**Authors:** Sara Pensieri, Roberto Bozzano, M. Elisabetta Schiano, Manolis Ntoumas, Emmanouil Potiris, Constantin Frangoulis, Dimitrios Podaras, George Petihakis

**Affiliations:** 1 National Research Council of Italy, Institute of Intelligent Systems for Automation, Genoa 16149, Italy; roberto.bozzano@ge.issia.cnr.it; 2 National Research Council of Italy, Institute of Marine Science, Genoa, 16149, Italy; elisabetta.schiano@ismar.cnr.it; 3 Hellenic Centre for Marine Research, Institute of Oceanography, Heraklion Crete, 71003, Greece; mntou@hcmr.gr (M.N.); mpotiris@hcmr.gr (E.P.); cfrangoulis@hcmr.gr (C.F.); dimitris@hcmr.gr (D.P.); gpetihakis@hcmr.gr (G.P.)

**Keywords:** dissolved oxygen, fluorescence, turbidity, intercomparison, operational oceanography, marine technology, ocean observing system

## Abstract

In European seas, ocean monitoring strategies in terms of key parameters, space and time scale vary widely for a range of technical and economic reasons. Nonetheless, the growing interest in the ocean interior promotes the investigation of processes such as oxygen consumption, primary productivity and ocean acidity requiring that close attention is paid to the instruments in terms of measurement setup, configuration, calibration, maintenance procedures and quality assessment. To this aim, two separate hardware and software tools were developed in order to test and simultaneously intercompare several oxygen probes and fluorometers/turbidimeters, respectively in the same environmental conditions, with a configuration as close as possible to real *in-situ* deployment. The chamber designed to perform chlorophyll-*a* and turbidity tests allowed for the simultaneous acquisition of analogue and digital signals of several sensors at the same time, so it was sufficiently compact to be used in both laboratory and onboard vessels. Methodologies and best practice committed to the intercomparison of dissolved oxygen sensors and fluorometers/turbidimeters have been used, which aid in the promotion of interoperability to access key infrastructures, such as ocean observatories and calibration facilities. Results from laboratory tests as well as field tests in the Mediterranean Sea are presented.

## 1. Introduction

A major task of operational oceanography is to produce and disseminate data that is of use to a great variety of applications. A number of services feeding from this data have already reached a satisfactory level of maturity, e.g., weather routing, sea state, hydrodynamic, search-and-rescue and oil-spill forecasts. An increased number of biochemical parameters of a certain quality should be incorporated in the observing systems and systematically monitored to extend the number of applications in which oceanographic data can be utilized and to improve our understanding of the driving mechanisms.

Sustained observations of biochemical variables, both at regional and global levels and over longer time-scales, are a key factor in undertaking operational oceanography goals, as set by scientific and societal needs [1,2]. Long-term, multi-parametric observations throughout the water column are a powerful means of studying the ocean as they can shed light on the driving forces of biochemical processes [3,4,5].

Also, model outputs may be improved through assimilation and/or validation procedures [6,7], enhancing our ability to predict the ocean state. Another advantage is that data availability at the monitoring sites of fixed-point observatories makes these areas suitable for inter-disciplinary studies that exploit more than one type of observatories [8,9,10]. In such cases, the limitations of data sets derived from different platforms can be identified [11], whereas their comparison can increase our confidence in the analyses results [12].

During the last decade, several European research projects (MFSTEP, ESONET, HYPOX, EMSODEV, ENVRIPLUS, FixO3, FERRYBOX, JERICO, to name a few) focused on the improvement of the monitoring of parameters related to marine biochemistry, ecology and climate change. This effort evolved in conjunction with the integration/harmonization of the inhomogeneous coastal/open-sea observing systems. A challenging aspect of achieving this objective is to produce inter-comparable biogeochemical measurements with the necessary accuracy in the long-term [13].

Although noteworthy attempts have been made in recent years to build up a common base of technical procedures and best practice for collecting data in the oceans, significant heterogeneity still exists all over Europe. At the broader international level, a more organic effort and long-term vision (25-year-plus time period) was made with the Ocean Observatories Initiative (OOI) [14], an integrated expandable infrastructure of science-driven platforms and sensor systems to measure physical, chemical and biological properties from the seafloor to the air–sea interface.

Technological design of observing systems, measured parameters, maintenance and quality control methodologies, as well as quality standards for sensors and data exchange, have not yet been standardized, at least for a number of important biochemical parameters.

In recent times, the expansion of ocean observatories has been often driven by specific or national interests and mainly undertaken through short-term research projects. Thus, the main challenge for the research community is to increase the coherence and the sustainability of these dispersed infrastructures by addressing their future within a shared international framework for ocean state monitoring [15].

In the context of a global observation system, measurements from Eulerian observatories not only complement other approaches but also contribute in a unique way. Such observations describe the full temporal behaviour of the system revealing, in many cases, a complex relationship among the measured variables [16,17]. The multi-parametric capability of Eulerian observatories provide a large number of correlated variables, enabling the contemporaneous advance of physical, biological, chemical and geological disciplines [18,19,20].

Regular calibration of sensors is one of the primary requirements for obtaining good quality data from ocean observatories and ensuring their long-term relevance as viable providers of reliable information on the marine environment. This is particularly true for observations of oceanographic variables leading oxygen consumption and primary productivity processes, as they require close attention of maintenance procedures, in terms of measurement setup and configuration [21].

Often, sensor calibrations made by a manufacturer and/or in a controlled environment provide very accurate measurements but this procedure inherently neglects real deployment conditions [22]. Among others, ambient characteristics (e.g., biologically active, corrosive, with a large range of variations of temperature, conductivity and pressure, and so on.) and technical constraints of the platform type in which the sensors have to be installed (e.g., sensor orientation, clamping methods, power availability, *etc.*) are the most relevant aspects to be taken into account. In some cases, for example for chlorophyll-*a* measurements, difficulties also arise due to the dominant native species which may differ from one geographical area to another.

The development of new time and cost effective tools for periodic intercomparison of biochemical sensors, to be used in an operational autonomous way by technicians taking care of the observatories, may avoid the necessity to regularly ship the sensors to manufacturers for post-deployment maintenance and calibration. This would reduce both the gap of collected time series, which is a key factor in long-term and permanent monitoring programs, as well as the associated costs.

In this paper, we describe two different innovative hardware and software tools to simultaneously intercompare dissolved oxygen sensors and fluorometers/turbidimeters in the same environment and the configuration characteristics of a real deployment.

The two designed tools are in compliance with the requirements of data quality resulting from several experiments aimed at defining the desired and the sufficient accuracy for dissolved oxygen measurements on existing platforms [23,24] and with the high-performance liquid chromatography (HPLC, hereafter) [25] method for chlorophyll-*a* data.

These tools were jointly developed by the National Research Council of Italy and the Hellenic Centre for Marine Research to be used in an easy way both in laboratory and on board ships during oceanographic cruises in order to facilitate the operational management of sensors deployed in ocean observatories.

The paper presents results of the intercomparison of several sensors carried out in the laboratory and the most recent data collected using some sensors deployed *in-situ*.

## 2. Materials and Methods

### 2.1. Hardware and Software Tools for the Intercomparison of Dissolved Oxygen Sensors

The aim of the experiment was to test an innovative tool for: the simultaneous intercomparison of several dissolved oxygen sensors to be used as best practice in the laboratories involved; and the operational management of marine observatories, even when not specifically equipped with metrological capabilities.

The overall instrumental setup included two Aanderaa Instruments AS (Bergen, Norway) optodes model 3975, four Sea-Bird Electronics, Inc. (Bellevue, WA, USA) model SBE43 sensors (SBE43, hereafter), one freshly calibrated conductivity and temperature recorder (Sea-Bird Instruments, Inc. model SBE37, s/n 5372) and two submersible pumps (Sea-Bird Instruments, Inc. model SBE5T and SBE5P).

SBE43 and optode instruments use different technology: the first is an electrochemical sensor mainly used for moored applications or to perform casts; and the second is an optic sensor characterized by high precision, thus very suitable for sensor calibration (see Appendix A for more details).

The hardware consisted of a chamber equipped with heater and circulator, an analogue and a serial acquisition board and a datalogging system. The software was based on specific routines to acquire and visualize the near-real time data provided by the oxygen probes to be tested and the ancillary reference measurements.

The chamber was constituted of a glass tank (800 mm × 500 mm × 500 mm) filled with 100 litres of filtered (mesh size 0.2 mm) sea water. Homogenous mixing in the tank was attained using the Haake N2 immersion circulator, whereas two aerators were switched on to increase oxygen concentration (introducing oxygen from the air into the water), or turned off to facilitate low dissolved oxygen concentrations. When needed, the top of the chamber was covered by a lid, leaving only a thin gap for the instrument cables in order to limit the exchange of oxygen between the atmosphere and the tank water. The Haake circulator also controlled the temperature inside the tank, allowing for fast (15 min) temperature stabilization, through a pump that circulated the water mass, via the heater and distributed back to the tank. During the experiment, the stability of the bath temperature was continuously monitored with a Sea-Bird Electronics, Inc. SBE35 Deep Ocean Standards Thermometer (accuracy ±0.001 °C) calibrated in a certificated primary standards laboratory.

A multipoint (eight steps) comparison in the range 4–6 mL·L^−1^ with Winkler samples was carried out in order to verify the performance of both optodes, since the sensor s/n 1647 was freshly calibrated by the manufacturer and never used after the calibration service (based on a robust multipoint calibration), whereas optode s/n 794 was an old sensor calibrated three years ago. Both optodes were validated before the experiment in 0% and 100% dissolved oxygen saturation points according to the manufacturer’s guidelines.

For each step of the intercomparison procedure, five water samples (replicates) were collected from the tank filling by siphoning calibrated oxygen bottles (~115 mL). This procedure was carried out at different controlled temperatures and by getting water as close as possible to the optodes. Different oxygen concentrations were obtained changing the temperature inside the bath and waiting for a complete stabilization of the water inside the tank (standard deviation of the temperature less than 0.01 °C before the water sampling). Oxygen stability in the tank was verified calculating the standard deviation of the acquired data during each step.

**Figure 1:**
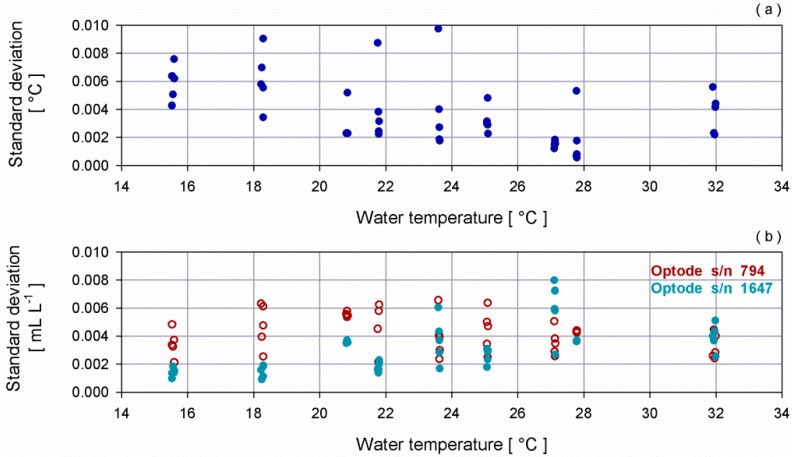
(**a**) Dispersion diagram between the temperature measured by the SBE35 and its standard deviation for each step; (**b**) dispersion diagram between the temperature measured by the SBE35 and the standard deviation of dissolved oxygen provided by optode s/n 794 (dark red empty dot) and optode s/n 1647 (dark cyan full dot).

Figure 1 shows the standard deviation of the temperature inside the bath measured by the SBE35 and the standard deviation of the dissolved oxygen acquired by the two optodes for each step during 10 min of measurements.

Water samples were taken with the recommended precautions to prevent any biological activity and gas exchanges with the atmosphere [26], while the standard procedure for Winkler requiring bottles to be over flown by three times their volume before fixation [27] was followed. Samples were maintained in the dark at the respective step temperature until analysis. Dissolved oxygen was determined within three hours after sampling using a Metrohm Dosimat 765, according to the Winkler method as modified by [28]. The method is considered to have an accuracy of <0.05 mL·L^−1^ [29,30], probably close to 0.01 mL·L^−1^ [31]. The overall analytical precision of the dissolved oxygen determination based on the replicates taken at seven out of eight steps was evaluated from the average standard deviation as <0.01 mL·L^−1^ (at the other step precision was 0.02 mL·L^−1^) demonstrating the high quality results of the analytic method.

**Figure 2:**
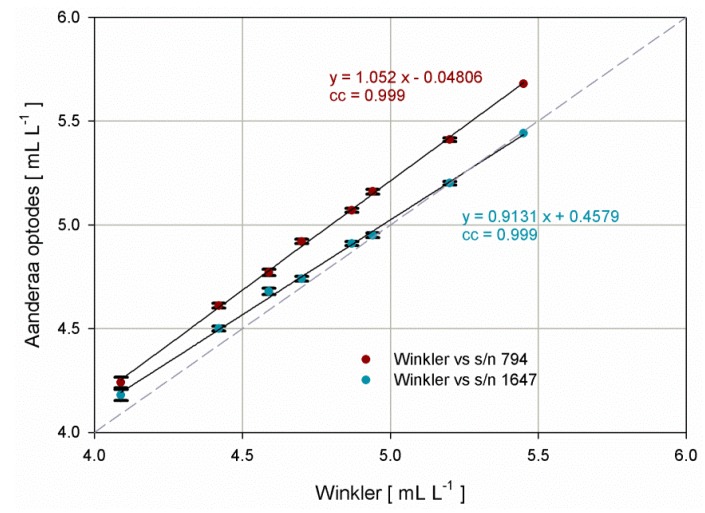
Dispersion diagram and linear regression between the results of the Winkler analytic method and the concentrations provided by two Aanderaa optodes model 3975 under analysis. Error bars represent the standard deviation, whereas dashed line represents the 1:1 relationship.

The direct comparison between the values obtained from Winkler titration and the two optodes positioned side by side showed a perfect agreement between optode s/n 1647 and a constant shift for optode s/n 794 (Figure 2).

Analysis on the performance of the optode measurements (O in Equations (1) and (2)) with respect to the Winkler (P in Equations (1) and (2)) were carried out by means of mean bias error and root mean square error, as defined in Equations (1) and (2) using J steps.

(1) $$\text{Bias}=\frac{1}{J}\sum_{j=1}^{J}\left(P_{j}-O_{j}\right)$$

(2) $$\text{Root Mean Square Error}=\sqrt{\frac{1}{J}\sum_{j=1}^{J}{\left(P_{j}-O_{j}\right)}^{2}}$$

The obtained bias and root mean square error were −0.2 mL·L^−1^ and 0.201 mL·L^−1^ for the optode s/n 794 and −0.0425 mL·L^−1^ and 0.057 mL·L^−1^ for the optode s/n 1647, respectively.

The satisfactory agreement between the Aanderaa optode s/n 1647 (OPT, hereafter) and the analytical dissolved oxygen concentration obtained through Winkler titration allowed us to consider OPT as a valid reference for the subsequent laboratory intercomparison tests of the four SBE43 sensors.

A specific software tool was developed to simultaneously acquire and store serial data from a Conductivity-Temperature-Depth (CTD) sensor and analogue voltage values provided by up to 8 dissolved oxygen sensors under testing within the tank. Measurements were collected by a datalogger, processed and displayed in real-time. The datalogger consisted of a computer running LabVIEW (a full-featured graphical programming language and development environment for embedded system design) and a National Instruments acquisition board (Austin, Texas, USA) (NI-8205) having multiple channel capability with adjustable voltage ranges and an accuracy of 0.15 mV in the range 0–5 V. When launched, the program configures the CTD sensor, then the user can in real time start to monitor the oxygen concentration measured by the sensors and can commence data logging and storing of CTD and raw analogue voltage data to separate text files. Data was collected at 1 Hz. Serial, data provided by the optodes were continuously collected by another computer that was synchronized in time with the data logger and the computer, collecting the output of the four SBE43 in tests.

### 2.2. IntercomparisonProtocol for Dissolved Oxygen Sensors

The protocol used to intercompare the four SBE43 oxygen sensors was based on the recommendation synthesized in [32] consisting in the analysis of the output of the sensors merged into the tank filled of water and the correspondent OPT data was used as a reference.

**Figure 3:**
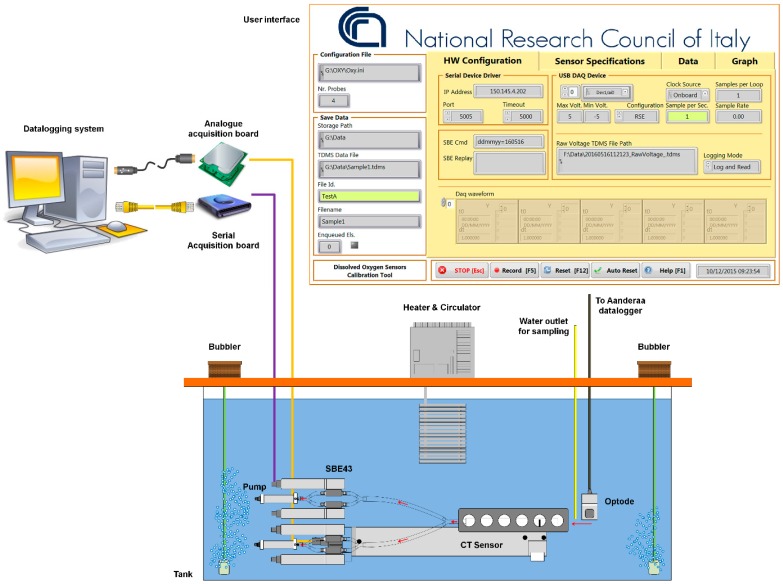
Sketch of the experimental setup used for the intercomparison of the dissolved oxygen sensors and user interface of the developed software tool.

Since the SBE43 is an in-line sensor, it has to be flushed with the same flow of the TC-cell which provides temperature and salinity measurements. Thus, four SBE43s were arranged in pairs, each pair flushed by a submersible pump (Figure 3). It must be noted that no antifouling methods were used at the intake and outlet of the duct.

A preliminary estimate of dissolved oxygen concentration in the tank was obtained in real-time applying the Owens-Millard [33] equation, using the raw analogue voltage output provided by four SBE43s, temperature and salinity measures from SBE37 and the manufacturer’s calibration coefficients since none of the sensors was deployed after their last calibration performed by Sea-Bird Instruments, Inc.

As pointed out in [34,35], dissolved oxygen sensors are flow sensitive. Mounting or positioning of the sensor with respect to the frame, or to the attached CTD, can affect the quality of the measurements, as well as give a slow response time to environmental changes. Thus, controlled calibration results can differ from real environment performances. This is especially true for SBE43 sensors that need to be flushed several seconds prior to measurement. For these reasons, the instruments under testing were assembled in configurations as close as possible to those employed in real deployments, and two different configuration setups were tested. In all trials, the tank with controlled temperature was used and the OPT was positioned as close as possible to the water intake of the dissolved oxygen duct.

Three SBE43s (s/n 2281, 2050, 1163) had a membrane 1 mil thick, suggested for moored applications, and one (s/n 541) had a thinner membrane (0.5 mil), which has a faster response and for this reason it is suggested for profiling purposes.

In the first test, the CT SBE37 was placed horizontally on the bottom of the tank and its outtake was connected using pipes and 1/2 inch Y-fittings to the two pairs of dissolved oxygen sensors; each pair was flushed by a submersible pump and was facing upwards.

In the second test, both the SBE37 and the pairs of oxygen sensors flushed by submersible pumps were horizontally suspended at the centre of the tank by means of ropes.

In order to verify that the chosen instrumental setup did not affect the performance of the SBE43 sensors, a preliminary test was performed sharply varying the oxygen concentration inside the tank. The flow rate effect was evaluated by measuring the flow rate of the two pumps in both chosen instrumental configurations and an average flow rate of about 33 mL·s^−1^ was measured for both pumps, really close to their nominal flow rate. Under this condition, it was possible to compare the time response with the one provided by sensor specifications, considering the time required to reach 99% of the final equilibrium, as stated by the manufacturer’s recommendations.

**Figure 4:**
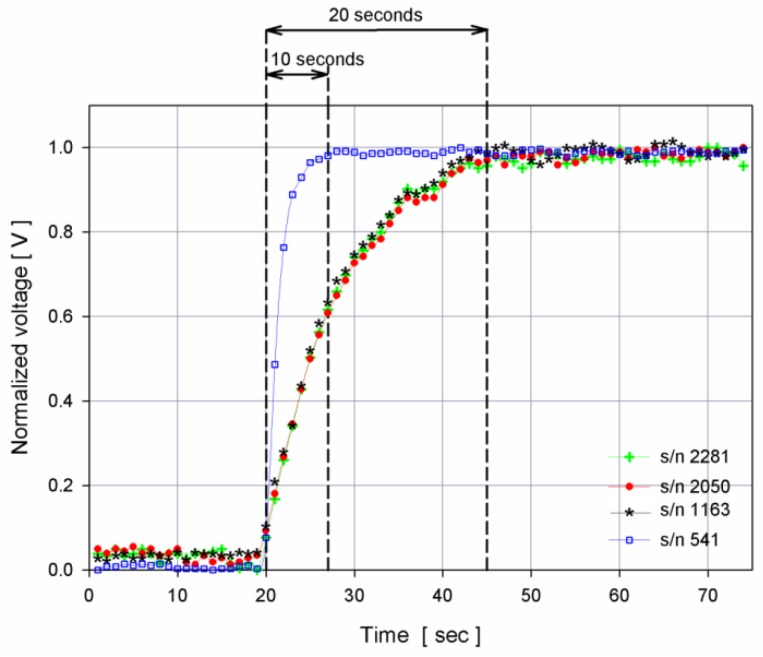
Response time of dissolved oxygen SBE43 sensors at a stabilized temperature of 15 °C. SBE43 DO s/n 541 uses a thinner membrane with respect to the other three sensors, hence it has a faster response to a sharp increase of oxygen concentration.

Figure 4 shows the response curves of SBE43 s/n 541 (0.5 mil membrane) and SBE43s s/n 2281, s/n 2050 and s/n 1163 (1 mil membrane) and their agreement with the SBE43 sensor specification that, at a temperature of 15 °C, shows a time response of about 15 s for an SBE43 with a 0.5 mil membrane and of about 23 s for a SBE43 with a 1 mil thick membrane. In case of a low flow rate, it is necessary to sample after a longer period of time in order to achieve the same performance.

### 2.3. Hardware and Software Tools for the Intercomparison of Chlorophyll-a and Turbidity Sensors

The designed hardware and software tools for chlorophyll-*a* and turbidity sensors intended to respond to the need for an easy intercomparison technique to obtain the same output from all sensors, simultaneously exposed to the same environmental conditions.

In order to fulfil the objective, an innovative ad-hoc chamber was designed. It was composed of a rectangular box (405 mm × 650 mm × 220 mm) with a lid having an internal volume of 28 L, with an inlet and an outlet from which a pipe passed through a magnetically coupled, centrifugal pump capable of a flow rate of 50 L·min^−1^. Cables from the sensors were passed out from the chamber by cable glands.

The chamber was designed to host sensors of different manufacturers characterized by serial and/or analogue output to perform an intercomparison exercise taking into account that, especially for turbidimeters of different brands, turbidity values can show discrepancies although measured on the same water sample [36,37].

Three combined fluorescence and turbidity sensors (model ECO-FLNTUS from WET Labs, Inc. (Philomath, OR, USA)) were available for the intercomparison. ECO-FLNTUS sensors are specifically designed to allow assessment of fluorescence and turbidity variability and interactions and provide excellent precision, reliability and overall performance for environmental monitoring applications [38]. Two of them (s/n 3372 and s/n 2776) were freshly calibrated by the manufacturer and never deployed at sea, one (s/n 615) was an aged sensor recently refitted by the manufacturer to measure chlorophyll-*a* in the range 0–25 µg·L^−1^ instead of 0–50 µg·L^−1^. The ECO-FLNTUS s/n 3372 was the only sensor with a measurement range of 0–50 µg·L^−1^.

All sensors had analogue and serial output capabilities with 4000-count range and an integrated anti-fouling bio-wiper that was removed before the test.

Recorded noise level (standard deviation of one minute of collected data) during repeated measurements was analyzed in order to evaluate the performance of the chamber in terms of stability. The test was carried out using different concentrations of 244 million of mL of *Chlorella* culture per part. The *Chlorella* culture was prepared by Aqualabs of the Institute of Marine Biology, Biotechnology and Aquaculture (IMBBC) of the Hellenic Centre for Marine Research (HCMR). *Chlorella* belongs to the phylum Chlorophyta and it is one of the most common algae in the Mediterranean Sea. The same test was performed using different solutions of disodium salt form of fluorescein (Na-fluorescein, also known as Uranine). Uranine is a synthetic organic compound slightly soluble in water and alcohol widely used as a fluorescent tracer in many applications.

**Figure 5:**
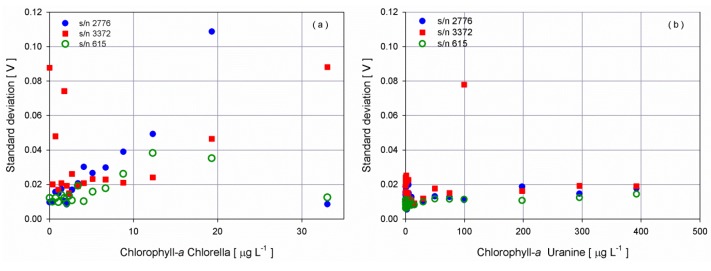
Noise level in terms of standard deviation of the fluorometers’ response as the concentration of (**a**) *Chlorella* and (**b**) Uranine increases.

Fluorometers results proved a good stability of the chamber with an average standard deviation of 0.0256 V and 0.0136 V during 1 min of measurements at 2 Hz for *Chlorella* and Uranine measurements, respectively. The noise was lower during the test performed with Uranine and this can be explained considering that the dye solution, being synthetic, is more stable with respect to *Chlorella* solutions (Figure 5). The same procedure was followed with the turbidimeter sensors and an average of 0.0260 V was measured, very similar to the ones retrieved for the fluorometers.

**Figure 6:**
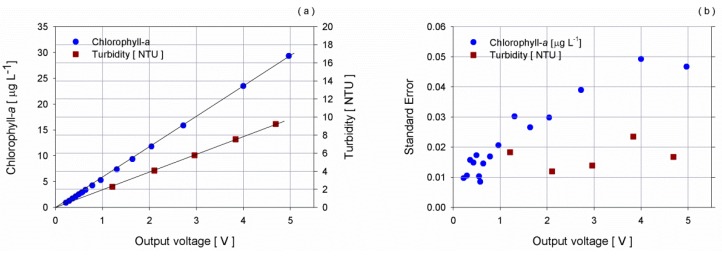
(**a**) Responses of fluorescence and turbidity sensor s/n 2776 and (**b**) corresponding standard error as the concentration of *Chlorella* (dot) and Formazine (square) increases.

The appropriate mixing of the chamber was tested verifying the linearity of the response of the fluorescence and turbidity sensor s/n 2776 to a progressive addition of *Chlorella* and Formazine, respectively (Figure 6). Formazine is the most common standard reference for the laboratory calibration of turbidimeter. In this test, the calibration coefficients provided by the manufacturer were used.

A software programme written in LabVIEW was specifically developed for managing an array of up to 5 ECO-FLNTUS sensors. A configuration text file contains for each instrument, an identifier, the specifications and the configuration commands. When launched, the program configures each sensor, then the user can start the acquisition phase. In order to avoid any interference between them, sensors are operated sequentially. Through a user interface, the operator can monitor in real-time, the average and the standard deviation of the sensor under test and when needed, can start to log and store the converted decimal outputs and raw analogue voltage signals from the instruments. Acquisition can last for a predefined time period or can be stopped by user. For each test point, the user can input a tag that will be included into the filenames of the ASCII files containing the acquired data. Analogue raw voltage signals from the sensors were acquired by a data acquisition board (National Instruments Inc., model NI-8205), whereas serial communication was performed through a serial device driver directly connected to the computer. For the turbidity test, a bent rubber sheet placed in front of the sensor array was used to suppress multiple reflections (if any) from the walls of the box.

### 2.4. IntercomparisonProtocol for Fluorometers/Turbidimeters

The adopted protocol dealt with the direct comparison between the output of the sensors and reference measurements that were constituted of different concentrations of 244 million cells per mL of *Chlorella* culture and known concentrations of Uranine solution for the fluorometers; and known concentrations of Formazine for the turbidimeters.

The ECO-FLNTUS fluorometer measures chlorophyll-*a* content at 470 nm and two bright blue LEDs (centred at 455 nm and modulated at 1 kHz) provide the excitation source. A blue interference filter is used to reject the small amount of red light emitted by the LEDs. The blue light from the sources enters the water volume at an angle of approximately 55°–60° with respect to the end face of the unit. Fluoresced light is received by a detector positioned where the acceptance angle forms a 140° intersection with the source beam. A red interference filter is used to discriminate against the scattered blue excitation light. The red fluorescence emitted is synchronously detected by a silicon photodiode (see Appendix B, for more details).

WET Labs Inc. calibrates its sensors using 25 µg·L^−1^ culture of *Thalassiosiraweisflogii* and highly recommends calibrating the fluorometers in field, by using native species of the area of the deployment. For this reason, the sensors were exposed to several concentrated solutions of *Chlorella*.

**Figure 7:**
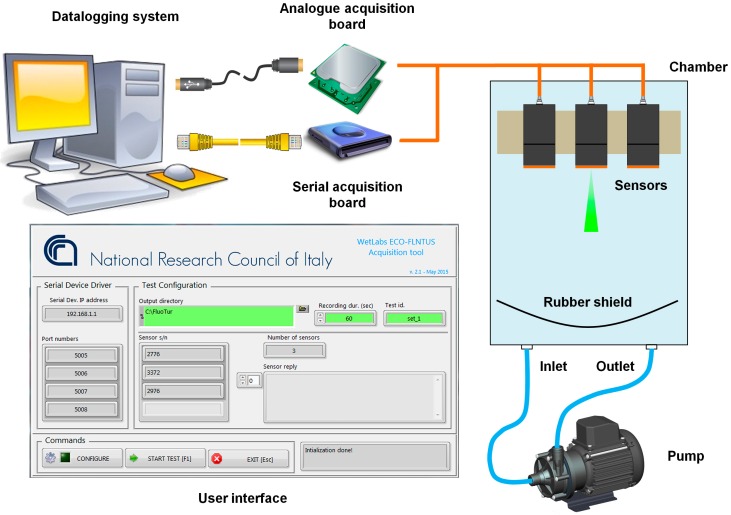
Sketch of the experimental setup used for the intercomparison of chlorophyll-*a* and turbidity sensors and user interface of the developed software tool.

The developed chamber was filled with sterile sea water and the three sensors were placed in line (Figure 7). A pumped circuit was activated for 30 s to mix the water inside the chamber before the measurements and was switched off during the measurement.

Sixteen steps were performed, comparing both voltage and raw count data provided by the three fluorometers.

The instrument response was monitored through a real-time plot and the online calculation of the average and the standard deviation of the voltage signal was visualized in the user interface. When the signal was considered stable enough, analogue voltage outputs were acquired sequentially from each sensor for 1 min at a sampling rate of 2 Hz. At the same time, the instrument stored raw data in the internal memory, the content of which was retrieved and deleted through the serial link.

Two replicates of three samples that corresponded to three levels of concentration (low, medium, high) were withdrawn from the chamber, filtered through GF/F filters under low vacuum pressure (<150 mmHg). These samples were analyzed through HPLC by the Chemistry laboratory and the Environmental and Analytical Chemistry laboratory of IMBBC of HCMR.

For the HPLC method, filters were immediately extracted in 3 mL of 100% acetone overnight and all sample extracts were analyzed by an HPLC system (Agilent Infinity 1260) comprising by Chemstation software, a degasser, a binary pump, an autosampler, a column thermostat and a diode array detector. The pigments were separated and quantified following the method described by Van Heukelem and Thomas [39]. For fluorometric measurements, extraction was performed in 10 mL of 90% acetone solution at 4 °C overnight and measurements were performed with a TURNER TD700 fluorometer. Both the aforementioned analyses were carried under dim light and low temperature conditions to minimize pigment destruction.

## 3. Results

### 3.1. Laboratory Tests for Dissolved Oxygen Sensors

**Figure 8:**
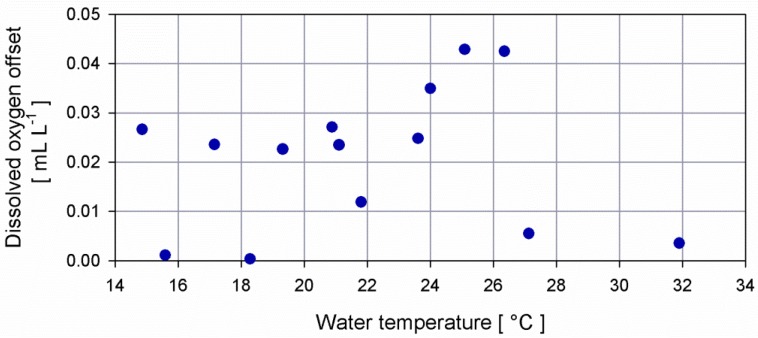
Absolute difference of dissolved oxygen measurements provided by the OPT positioned close to the inlet of the duct and the optode s/n 794 suspended at the outlet of the pump.

During the whole experiment for the dissolved oxygen intercomparison, 14 steps were performed with a temperature range from 15 °C to 32 °C and salinity spanning from 38.8 psu to 39.2 psu. For each point, homogenous mixing in the tank was attained using the immersion circulator and verifying the congruity of the measurements provided by the OPT, positioned close to the inlet of the duct and the other optode (s/n 794) suspended at the outlet of the pump. Two aerators were switched on to increase oxygen concentration or turned off to facilitate low, dissolved oxygen concentrations. Sea water was refrigerated during the night and progressively warmed during the day to reach the desired set points in temperature. Temperature in the laboratory was set at the same temperature of the water by means of an air conditioner for the whole experiment duration. Temperature and salinity were left to stabilize with a standard deviation, on average equal to 0.0058 °C for temperature and of 0.0020 psu for salinity. In terms of oxygen, the water inside the tank was left to homogenize and saturation was measured by both OPT and optode to reach 100% or higher, then 5 min of continuous raw voltage measurements by each SBE43 were collected and stored. Figure 8 shows the difference of the dissolved oxygen measured by OPT and optode 794 during each step.

Comparison between all SBE43 sensors and the OPT indicates a slight overestimation of all SBE43 independently from the type of membrane (for profiling or moored applications) although linearity is maintained. The root mean square error and the bias, calculated using Equation (1), show a misfit between the reference OPT and the SBE43 sensor of about 0.32 mL·L^−1^ and 0.29 mL·L^−1^ on average, respectively.

Both configurations used in the first test (SBE37 in an horizontal position and two pairs of SBE43 and pump in a vertical position) and in the second test (SBE37 and two pairs of SBE43 and pump in an horizontal position) showed the same results, indicating that the orientation of the SBE43 with respect to the SBE37 is irrelevant, provided that it is flushed with the same stream of the CT-cell and the flow rate of the pump is large enough.

**Figure 9:**
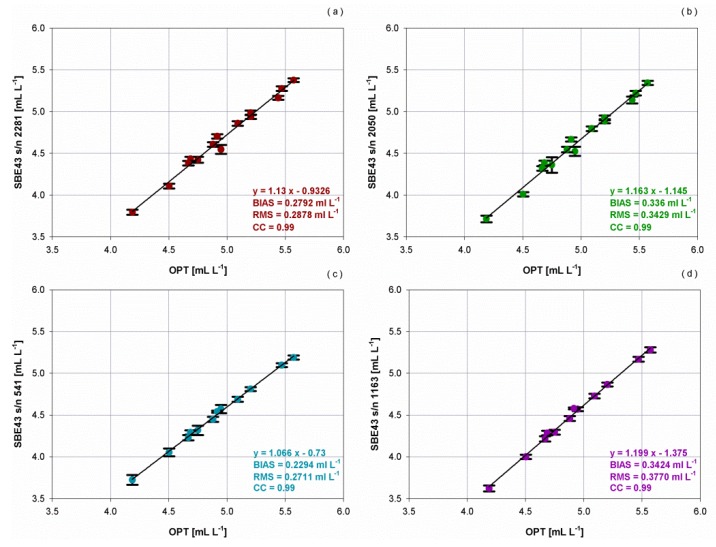
Dispersion diagrams of OPT against the SBE43 sensors (**a**) s/n 2281, (**b**) s/n 2050, (**c**) s/n 541, (**d**) s/n 1163 before (full dot) and after (empty square) the SOC correction. Error bars correspond to five times’ the measured standard deviation.

Results revealed the need to adjust the linear slope scaling coefficient (SOC) of SBE43 sensors: to this aim, the correction ratio (CR) between the OPT measurements and the corresponding concentrations provided by the SBE43 was calculated. The updated values of SOC were obtained multiplying SOC and CR values. Figure 9 summarizes the performance achieved using old and updated SOC.

The use of new SOC allowed an improvement in terms of mean bias error and root mean square error of 0.21 mL·L^−1^ on average.

The analysis of the performance of the oxygen sensors cannot be separated from the fact, that a small deviation of the oxygen concentration from fully saturated water mass, can strongly influence the air-sea oxygen flux [40,41]. Furthermore, the comparison between the theoretical saturation values of the water at a predefined temperature and salinity and the measured percent saturation, is an indicator of the performance. Two separate experiments were carried out to evaluate the performance of the new curves, with respect to temperature and salinity variations in the most common range of a real deployment in the Ligurian or Cretan Sea. In the first one, temperature was increased from 15 °C up to 32 °C at a constant salinity of 39 psu whereas the second test was carried out at a constant temperature of 26 °C and salinity, increasing from 34 psu up to 39 psu.

During the performed steps with constant salinity and different temperatures, very good agreement was found between the OPT measurements and theoretical values at 100% saturation (average bias of 0.018 mL·L^−1^). Underestimation up to 13% in saturation were evidenced for the SBE43 sensors that showed lower values for all tests.

Also keeping the temperature constant and varying the salinity, the same good agreement between OPT measurements and theoretical values at 100% saturation was observed, obtaining an average bias of 0.067 mL·L^−1^. Simultaneously, all SBE43 sensors were not fully saturated, having an average difference with respect to OPT, of about 5% for s/n 541 and 1163 and of 1% for s/n 2281 and 2050.

OPT saturation values for all steps and for constant temperature and salinity tests were compared to the percentage of oxygen saturation provided by SBE43 sensors, before and after the SOC adjustment. Percentage of oxygen saturation for SBE43 was calculated as the ratio between the measured oxygen concentrations and the theoretical oxygen solubility, defined as the volume of oxygen gas at standard temperature and salinity conditions absorbed from humidity-saturated air, at total pressure of one atmosphere per unit of volume of the liquid, at the temperature of measurement.

**Figure 10:**
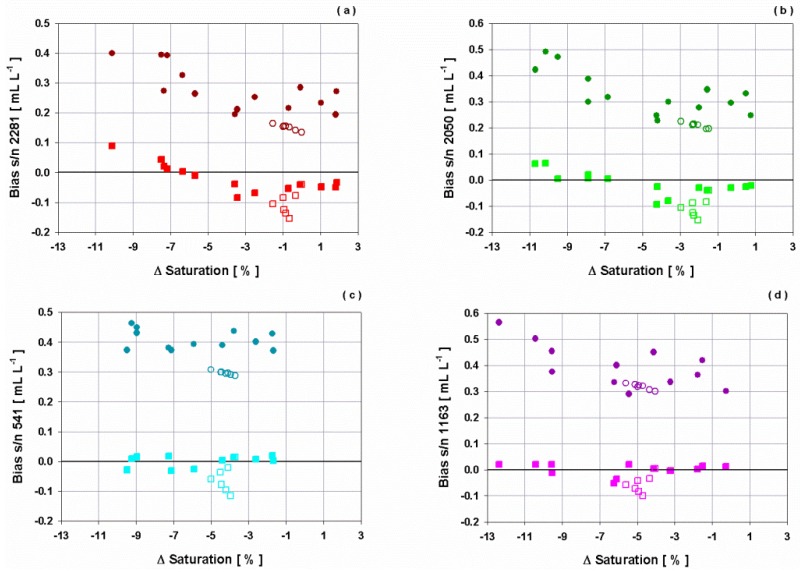
Bias of dissolved oxygen concentration with respect to saturation offset for SBE43 sensors (**a**) s/n 2281, (**b**) s/n 2050, (**c**) s/n 541, (**d**) s/n 1163 before (dot) and after (square) the SOC adjustment at constant salinity and varying temperature (full marker) and at constant temperature and varying salinity (empty marker).

Results show that by varying the temperature, the offset between the dissolved oxygen concentration measured by the OPT and the SBE43s before the comparison decreases as the saturation gap reduces, whereas very close values were obtained after the SOC adjustment independently from the gap in oxygen saturation. In isothermal conditions, the increase of salinity did not affect the measurements and the constant offset that was observed for all SBE43 sensors before the SOC adjustment was reduced by applying the obtained curves (Figure 10).

### 3.2. Laboratory Tests for Chlorophyll-a Sensors

Analysis using the HPLC method has to be considered as standard for the assessment of fluorometers’ performance, although it requires expertise and specifically equipped chemical laboratories, not commonly found in institutions for managing operational marine observatories.

The output of the ECO-FLNTUS fluorometer has a linear correlation with chlorophyll-*a,* and concentration can be calculated as:

(3) Concentration [µg·L^−1^] = (OUT − BLANK)·SF

where concentration is the chlorophyll-*a* concentration of the sample of interest, OUT is the raw measured output, BLANK corresponds to the blank value, that is the clean water offset and SF is the scale factor. Equation (3) is valid for both voltage and count outputs.

The determination of the blank value is one of the most difficult procedures in the calibration routine [42]. Its variability can negatively affect the measurements, especially when chlorophyll-*a* observations are carried out in oligotrophic basins as in the case of the Cretan Sea, where measured values are close to the blank value.

Several tests were performed considering the output of the instrument (both voltage and count) in air (AIR), using tap water (TW), filtered sea water (FSW) and a tape (TAPE) on the detector in order to verify the impact of BLANK value on the estimates of chlorophyll-*a* concentration. One minute of simultaneously raw voltages at 2 Hz and counts at 1 Hz were acquired, after the stabilization of the values at a controlled temperature of 22 °C.

Measurements in air and with the detector window covered by tape show very similar values, whereas concentrations are greater for filtered sea water and tap water, with respect to the dimming of the window of the detector. Especially in the case of tap water, turbidity can be responsible for the increase of the BLANK due to the step up of light scatter [43].

Filtered sea water could be considered as the appropriate blank value, only if the removal of phytoplankton culture is made in the same ambient irradiance of the *in-situ* environment. Thus, according to the manufacturer's advice, the most accurate blank value can be obtained by completely covering the window of the detector with black tape and leaving the window of the emitter uncovered [44].

Table 1 summarizes the difference of chlorophyll-*a* concentration in unit of µg·L^−1^ using different BLANK values (air, filtered sea water, tap water) in voltage, with respect to the BLANK value obtained with the detector covered by the black tape.

BLANK value corresponding to the BLANKTAPE was used in the experiment.

The intercomparison between the three fluorometers was performed at a controlled temperature of 22 °C, the same as the calibration sheet provided by the manufacturer. The chamber was filled with filtered sea water and sixteen steps were performed adding small aliquots of *Chlorella* culture to increase progressively the concentration of chlorophyll-*a* in the chamber. The readings for a period of one minute, after checking that the standard deviation was less than 0.02 V, were recorded. Samples were mixed through a magnetically coupled centrifugal pump, to achieve homogenous solutions. Three samples of different concentrations corresponding to a low, medium and high concentration for the Mediterranean Sea were collected from the chamber, in order to measure chlorophyll-*a* concentration through a laboratory fluorometric instrument and HPLC.

Results between the two different laboratory techniques show an average standard deviation of 0.16 µg·L^−1^, with a maximum of 0.37 µg·L^−1^ for the highest chlorophyll-*a* concentration.

To calculate new scale factors, HPLC results were used as reference and both voltage and count outputs were considered.

Factory calibration scale factors are greater than those computed from experimental data, for the two ECO-FLNTU with a range of 0–25 µg·L^−1^: nominally they are 6 µg·L^−1^ V^−1^ instead of 4.19 µg·L^−1^ V^−1^ for s/n 2776 and 4.25 µg·L^−1^ V^−1^ for s/n 615 resulting from the experiment. A scale factor value of 9.14 µg·L^−1^ V^−1^ close to the nominal 10 µg·L^−1^ V^−1^ was found for the fluorometer with an extended range (s/n 3372).

**Figure 11:**
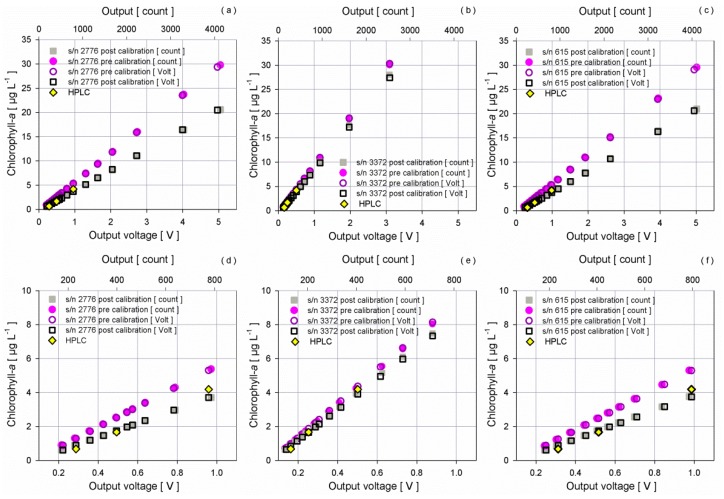
Dispersion diagrams of chlorophyll-*a* concentrations and output of the fluorometers in voltage (empty marker) and count (full marker) before (circle) and after (square) the experiment, for fluorometers (**a**,**d**) s/n 2776, (**b**,**e**) s/n 3372, (**c**,**f**) s/n 615. Diamonds correspond to HPLC result. Top diagrams refer to the entire voltage range; bottom diagrams are a zoom in the range 0–1 V.

Figure 11 shows the scatter plots between the concentration of chlorophyll-*a* estimated using manufacturer and new values for BLANK and SF in Equation (3) and the instrument response of the three sensors (voltage and count).

Results confirm the constant ratio between voltage and raw count, showing the linearity of the sensors throughout the entire voltage range as well as the very good correlation between the new curves and the reference samples analysed through HPLC method. The perfect agreement between voltage and count proves the reliability of using, in an operational way, the voltage provided by the fluorometers when they are installed on a multiparametric device (*i.e.*, SBE16plus) without the necessity to consider the raw count values. The impact of the new curves on the concentrations is relevant for both low and elevated chlorophyll-*a* values. In this study, the curves provided by the manufacturer show always an overestimation of the concentrations that can be quantified with an absolute bias of 0.59 µg·L^−1^ and 0.58 µg·L^−1^ for the two sensors with a narrow range (0–25 µg·L^−1^) and of 0.12 µg·L^−1^ for the fluorometer with an extended range (0–50 µg·L^−1^).

The error is exponential with the increase of the voltage and at the maximum voltage output, it is greater than 8 µg·L^−1^ for the two narrow range fluorometers and about 3 µg·L^−1^ for the extended range sensor using the manufacturer curves, with respect to the application of the new curves.

To investigate the relationship between the instrument response to *Chlorella* culture and synthetic dye, Uranine was used. The produced fluorescence of synthetic dyes and *Chlorella* show a peak excitation at 494 nm and 440 and nm and a peak emission at 521 nm and 680, respectively. Although the fluorescence produced by Uranine takes place at a different wavelength and only a tail of its fluorescence peak could be detected by the ECO-FLNTUS, Uranine and Basic Blue 3 (BB3) are commonly used to assess fluorometer performance [45].

Starting from a solution having a high concentration (20 mg·L^−1^) of Uranine, different volumes were progressively poured into the chamber in order to get different increasing fluorescence concentrations. The experiment was performed at a controlled temperature of 22 °C and within one day to avoid loss of stability of the dye solution.

**Figure 12:**
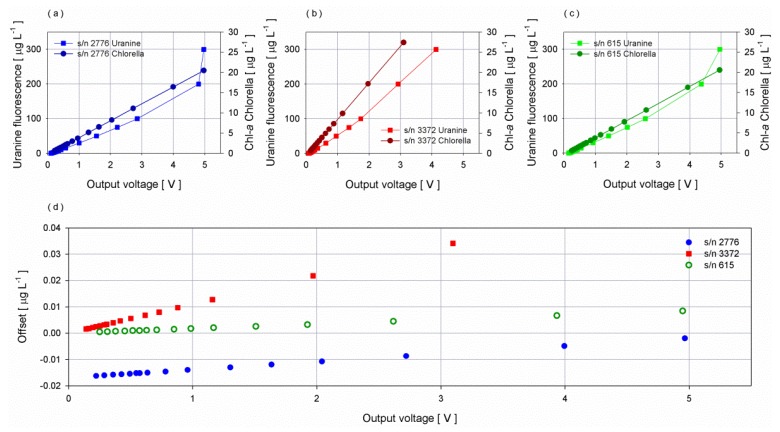
Dispersion diagrams of chlorophyll-*a* concentrations of *Chlorella* and fluorescence of Uranine *vs.* fluorometers response for sensor (**a**) s/n 2776, (**b**) s/n 3372 and (**c**) s/n 615. (**d**) Offset between the chlorophyll-*a* estimates using the shifted curve from the dye solution and the *Chlorella* concentration.

Fluorescence shows a linear response with the increasing of the dye solution up to 2.5 V: beyond this threshold a small drift was observed. Sensors with narrow range saturated in the last two steps, corresponding to concentrations of Uranine greater than 200 µg·L^−1^ (Figure 12). Comparing the two curves obtained using *Chlorella* and Uranine, an angular shift of 11.81 degree for both the sensors with reduced range and of 5.33 degree for the sensor with extended range was observed. To evaluate the feasibility of using Uranine instead of *Chlorella* for operational periodic intercomparison, an error analysis was performed taking into consideration the obtained angular shift. Specifically, Uranine curves were rotated to the above mentioned angles and compared to *Chlorella* curves. The difference between the chlorophyll-*a* concentrations (obtained applying the *Chlorella* curve and the rotated Uranine curve) was considered as an error. Results show an increasing of the error with the increase of the output voltage for all sensors spanning from −0.020 µg·L^−1^ to 0.040 µg·L^−1^, thus falling within the average error obtained and considering the standard deviation of one minute of collected data (Figure 12).

The comparison between the two curves illustrates the feasibility of using Uranine to compare fluorometers considering the angular shift, provided that the pH of the water filling the chamber is above 8. Indeed, several studies have been performed to verify the influence of pH variations on the fluorescence of the Uranine [46,47,48], and results showed an increase of the fluorescence for acid solutions and constant values from neutral up to strongly basic solutions.

To verify the impact of pH variations on fluorescence properties of Uranine using the developed instrumental setup, twenty-nine measurement points were performed with pH spanning from 3.5 up to 11.

Starting from the chamber filled with filtered sea water at 8.64 pH, a volume of 30 mL Uranine stable solution (20 mg·L^−1^) was added and aliquots of NaOH 0.5 M and HCl 0.5 M were progressively poured in the chamber to adjust pH. A submersible pump was switched on to mix the water volume at each step and off during the data acquisition, to ensure a homogenous mixing of the water volume. Latch HQ40d portable meter was used to monitor pH.

The same software tool used for the fluorescence experiment was employed to collect raw data in real time from the three ECO-FLNTUS sensors.

**Figure 13:**
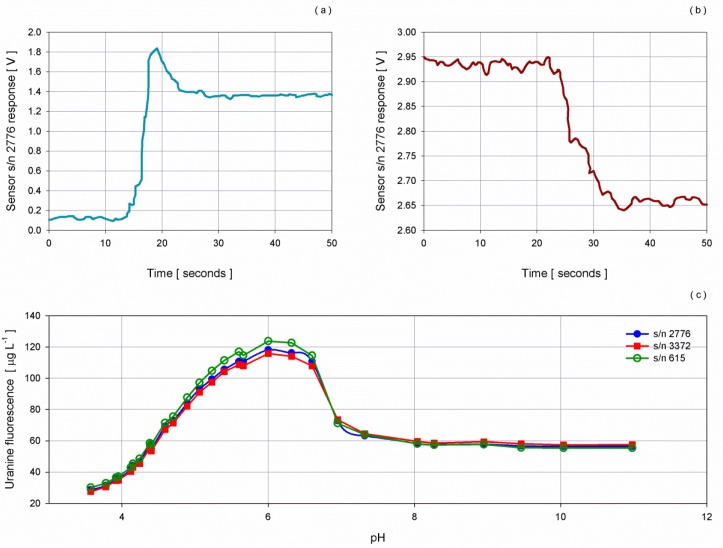
Voltage signal of sensor s/n 2776: (**a**) the steep increase when 30 mL of Uranine was poured into filtered sea water and; (**b**) the rapid drop when 1 mL of HCl 0.5 M was added into the chamber making pH decreasing from 4.7 down to 4.4.; (**c**) the chlorophyll-*a* curves as pH varies.

Figure 13 shows an example of the response of the fluorometer at the beginning of the test when 30 mL of Uranine was poured into filtered sea water causing a steep increase in the acquired voltage and when 1 mL of HCl 0.5 M was added to decrease pH, thus producing a drop in the signal.

Chlorophyll-*a* concentrations confirmed the non-linear behaviour for acid solution, especially for pH values below 7 and quite constant values for pH exceeding 8, pointing out the feasibility to inter-compare/inter-compare fluorometers using the Uranine dye solution in a controlled environment with pH greater than 8.

### 3.3. Laboratory Tests for Turbidity Sensors

ECO-FLNTUS turbidimeter has a linear response throughout the measurement range that can be described by

(4) Turbidity [NTU] = (OUT − BLANK)·SF

where Turbidity is the level of turbidity of the sample of interest, OUT is the raw measured output, BLANK corresponds to the clean water offset and SF is the scale factor. Equation (4) is valid for both voltage and count outputs.

For the experiment, the same combined fluorescence and turbidity sensors used for the chlorophyll-*a* tests were used. Two of them (s/n 615, s/n 2776) have a measurement range of 0–10 NTU whereas s/n 3372 has a wider range of 0–25 NTU.

To determine the BLANK values of all three sensors, the same procedure followed for the chlorophyll-*a* test was used. Several tests were performed by considering the output of the sensor in air (AIR), using filtered sea water (FSW) and filtered sea water with a black rubber shield inside the chamber (FSWB), with a tape (TAPE) on the detector. One minute of simultaneously raw voltage at 2 Hz and count at 1 Hz were recorded after the stabilization of the values at a controlled temperature of 22 °C.

Measurements in air show the greatest differences, with respect to the results obtained with the tape on the detector, whereas the offset between observations in filtered sea water, without and with the black rubber shield in front of sensors, evidenced the importance of avoiding reflection inside the chamber (Table 2). Measured and manufacturer BLANK show very similar values for the output in voltage, whereas greater values were obtained for count data even if the ratio between the output in voltage and count for the three tested sensors remains almost constant.

As for the chlorophyll-*a* experiment, filtered sea water could be considered an appropriate blank only if the removal of phytoplankton culture is performed in the same irradiance condition of the *in-situ* environment. For this reason, in compliance with the manufacturer's advice, the BLANK value used in the experiment was defined as the BLANKTAPE value.

The intercomparison between the three turbidimeters was performed as for the fluorometers test, at a controlled temperature of 22 °C, the same of the calibration sheet provided by the manufacturer. The chamber was filled with filtered sea water and seven steps were performed increasing the concentration from 2 NTU up to 12 NTU. Unwanted reflections were avoided by placing a sheet of black rubber in front of the sensors. To achieve homogenous solutions, the same magnetically coupled, centrifugal pump used for the chlorophyll-*a* test was employed.

Results show an almost perfect agreement between the three scale factors provided by WET Labs Inc. and the obtained values, pointing out that the developed new chamber is very suitable to perform intercomparisons of turbidimeters and in turn, of fluorometers.

Since the aim was to intercompare the sensors to be routinely installed in the Mediterranean basin, the scale factor to be operationally used was obtained considering a range between 0 NTU and 6 NTU. Within this hypothesis, the obtained scale factor was 2.06 NTU V^−1^ (0.002441 NTU count^−1^) for s/n 2776 and 2.005 NTU V^−1^ (0.002385 NTU count^−1^) for s/n 615, instead of 2 (0.0024 NTU count^−1^ and 0.0022 NTU count^−1^, respectively) from datasheet and 4.82 NTUV^−1^ (0.005926 NTU count^−1^) for s/n 3372 instead of 5 NTU V^−1^ (0.0061 NTU count^−1^).

**Figure 14:**
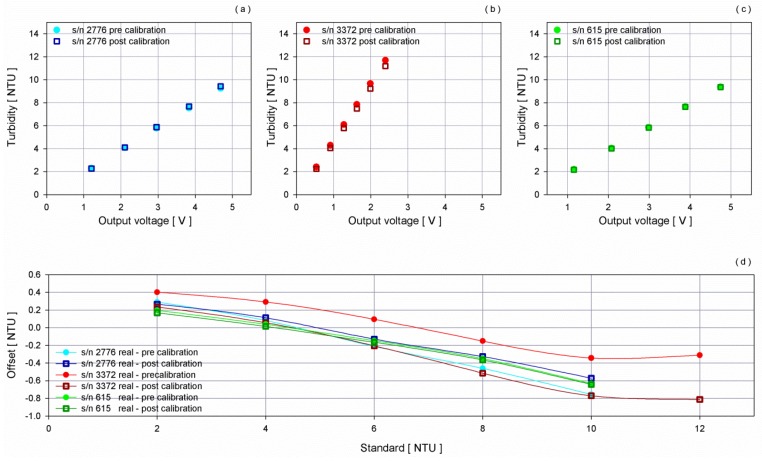
(**a**–**c**) Scatter plots of turbidity estimated by the three sensors before and after the experiment as instrument response in voltage varies; (**d**) Curves of the offset between the reference concentrations and the values obtained applying old and new curves.

Figure 14 shows the estimates obtained, applying both the factory and the new curves and their difference with respect to the standard concentration.

For the two sensors with the narrowest measurement range, the curve of the offset between standard concentrations and the values obtained, applying both old and new curves overlaps up to 6 NTU. A better fit to the reference data was also obtained for turbidity greater than 6 NTU with respect to the original calibration. The use of a maximum calibration standard of 12 NTU, corresponding to half range for sensor s/n 3372, resulted in a greater difference between the standard and the estimates obtained in applying the old and the new curves. Nonetheless, the new curves provide an almost perfect match for the measurements of the three turbidimeters in the range 0–6 NTU, which is of interest for deployment in the Mediterranean Sea.

## 4. Conclusions

Marine ecosystem dynamics are inherently non-linear, and resolving temporal and spatial variability in the oceans is a very difficult task. Understanding ocean processes is often limited by a lack of multidisciplinary oceanographic time-series datasets at appropriate spatial and temporal resolutions.

In the last few years, technological evolution has pushed forward the development of innovative automated sensors for the monitoring of key biochemical parameters.

Factory calibration can be inadequate taking into account operational constraints of installation of optical sensors into mobile or fixed platforms. Thus, it is fundamental to create tools which enable to pre- and post-compare sensors not necessarily in metrologically equipped laboratories, by using an instrumental setup as close as possible to real deployment conditions.

The developed, dissolved oxygen intercomparison tool allows for a continuous and real-time monitoring of both reference sensor and instruments to be tested and it needs to be valid under different conditions (stable or varying temperature and/or salinity) by providing great accuracy. Obtained time response from the tested SBE43 was coherent with the specifications pointing out that the tank and the tool could be useful to detect also quick changes in the dissolved oxygen concentration. The test performed, using two different installation methods (with measurement package in horizontal and vertical arrangement), evidenced that both setups can be operationally employed, since no differences were found in the results providing that the flow rate of the pump is fast enough (around 33 mL·s^−1^).

The possibility to acquire real time, raw voltage measurements from oxygen sensors without using a multiparametric probe, allows for the simultaneous intercomparison of several oxygen probes in a small bath. This has several advantages: it is easier to control and keep stable the water temperature and in turn, it is more practical to perform several steps in a short period of time thereby reducing the waiting time between the steps. Furthermore, the tool is easy to use both in laboratory and onboard ships during oceanographic cruises or surveys.

The designed chamber for the intercomparison of multiple fluorometers and turbidimeters allows easy and repetitive measurement steps, and it proves to be stable and to guarantee an appropriate mixing during repeated tests, with same concentrations of chlorophyll-*a* and turbidity.

The new software tool developed for the simultaneous intercomparison of several probes allows all tested sensors to be exposed to the same concentration of native phytoplankton species, to measure chlorophyll-*a,* avoiding problems due to the decay of the species. The chamber demonstrates versatility, to be used with both *in vivo* species and dye solutions, allowing tuned instruments to show similar responses if exposed to similar conditions.

Results demonstrate that an angular shift of the fluorescence/chlorophyll-*a* concentration curve exists when moving from a native species solution to a dye solution and that, after a first adjustment through HPLC, it is possible to use this shift to perform further comparisons of the same sensor only using dye solution (*i.e.*, Uranine, BB3, *etc.*), provided that the pH is not influencing the fluorescence properties of the dyes.

Results for turbidity coincide with the calibration performed by the manufacturer confirming the overall reliability of the new chamber for performing comparisons.

Furthermore, the two tools are really promising when shared within a network of observatories, as the Mediterranean Moored Multi-sensor Array (M3A) network [49] or the FixO3 network [50] for intercomparison of sensors, before and after the deployment, since the possibility of operating several sensors in parallel increases the quality of measurements and allows the detection of sensor failure or drift.

The use of these new tools could be a viable way of avoiding gaps or aberrations in time series data sets between two consecutive deployments and the enhancement of long term, *in situ* monitoring of marine biogeochemical parameters. Indeed, the used sensors were successfully deployed on the W1-M3A observatory [51] at the end of June 2015. A multiparametric probe equipped with the SBE43 s/n 2050 and the ECO-FLNTUS s/n 2776 was installed on the spar buoy at a 6 m depth. On 13 November 2015, the sensors were replaced with the SBE43 s/n 2281 and the ECO-FLNTUS s/n 3372.

At the end of August, an oceanographic cruise took place in the surroundings of the W1-M3A observatory and, on that occasion, CTD casts and water samples were collected. Winkler titration method applied to the water sample correspondent to a 6 m depth and the data provided by the cast show a good agreement with the measurements collected by the equipment on board the observatory.

**Figure 15:**
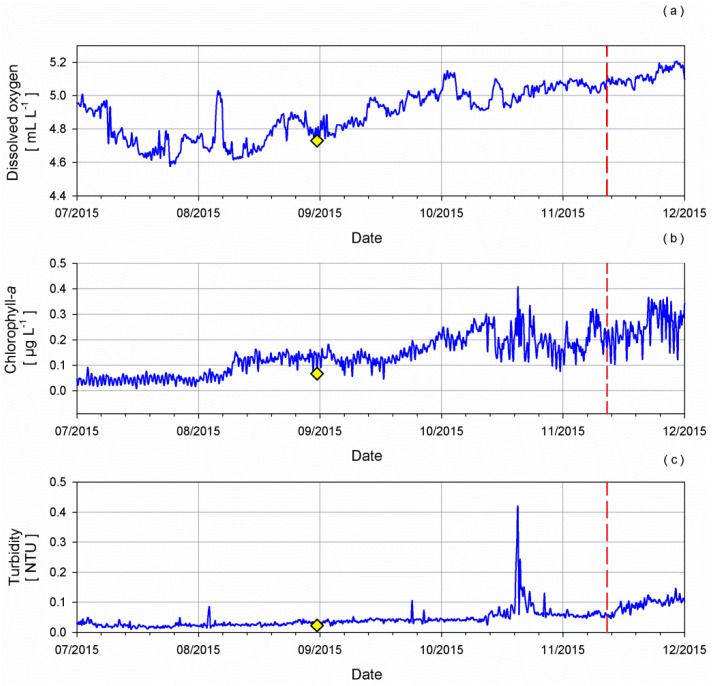
Time series of (**a**) dissolved oxygen, (**b**) chlorophyll-*a*, (**c**) turbidity acquired by the instruments installed on the W1M3A observatory at a 6 m depth. Long dashed lines correspond to the change of the sensors whereas diamonds show cast results.

Figure 15 shows, as an example, the acquired time series of dissolved oxygen, chlorophyll-*a* and turbidity from July 2015 to December 2015 at a 6 m depth.

The change of sensors on 13 November 2015 did not affect the continuity of the measurements. This demonstrates the usefulness of the intercomparison exercise carried out for the long-term monitoring of biochemical properties on oceanographic fixed platforms.

## Figures and Tables

**Table 1:** Difference of chlorophyll-*a* concentration in unit of µg·L^−1^ computed using different BLANK values (air, filtered sea water, tap water) with respect to using BLANK value obtained with the detector covered by the black tape.

Chlorophyll-*a* Offset		WET Labs, Inc. ECO-FLNTUS Sensors		
	s/n	2776	3372	615
SF·(BLANK_TAPE_ − BLANK_AIR_)		0.026	−0.034	0.023
SF·(BLANK_TAPE_ − BLANK_FSW_)		−0.356	−0.298	−0.417
SF·(BLANK_TAPE_ − BLANK_TW_)		−7.930	−8.207	−8.725

**Table 2:** Difference of turbidity concentration in NTU unit computed using different BLANK values (air, filtered sea water without and with a black rubber shield) with respect to using BLANK value obtained with the detector covered by the black tape.

Turbidity Offset		WET Labs, Inc. ECO-FLNTUS Sensors		
	s/n	2776	3372	615
SF·( BLANK_TAPE_– BLANK_AIR_)		–2.003	–2.169	–2.326
SF·( BLANK_TAPE_– BLANK_FSW_)		–1.497	–1.867	–1.597
SF·( BLANK_TAPE_– BLANK_FSWB_)		–0.404	–0.270	–0.290

## Appendix A: Dissolved Oxygen Sensors Technology

The technology for the dissolved oxygen monitoring in the ocean is nowadays mature and commercial sensors exploit two different methodologies: electrochemistry and optics.

In the first category, electrochemical sensors such as the Sea-Bird Electronics, Inc. model SBE43 are based on an upgraded version of the Clark polarographic membrane [52,53] developed by Clark [54] that measures the partial pressure of oxygen by means of the rate of the diffusion of oxygen through a membrane. Specifically, the Clark cell works on the principle of molecular O_2_ at a gold cathode: the oxygen diffuses across a membrane and it is converted to OH^−^ ion at the gold cathode, and oxygen concentration is proportional to the induced current.

SBE43 provides an estimate of oxygen dissolved into the water taking into account pressure and salinity [55] and applying the Owens-Millard equation [33].

SBE43 sensors need to be flushed to reach the level of accuracy and stability reported in the technical specifications and have an equilibration time from few seconds up to some tens of seconds, depending on the thickness of the membrane. They are suitable for moored applications, Conductivity-Temperature-Depth (CTD) sensor casts and mobile platforms since they can have a quick response to oxygen change at the expense of a loss of sensitivity, producing a predictable drift in the measurements with time.

In the second category, the most frequently used optical sensor for oxygen monitoring is the Aanderaa Instruments AS optode class of instruments whose measurements are based on the principle of fluorescence quenching of a platinum porphyrin complex immobilized in a sensing foil [56,57]. Chemicals in the foils are excited by a blue light and it is possible to obtain the quantity of dissolved oxygen from the shift in phase between the emission and the fluoresced red signal. The quenching is expressed by the Stern-Volmer equation [58] and the dependence on salinity and pressure is based on the Garcia and Gordon formula [55]. Optodes provide long term stability and high precision but are characterized by a slow response time that can affect measurements in the upper ocean where vertical gradients in temperature and oxygen may be significant. This type of sensor is implemented in Argo floats, gliders and on moorings and it is generally used as reference for laboratory calibration due to the accuracy in the measurements, although it must be noted that the best standard analytical method for discrete sampling is the Winkler method [27].

## Appendix B: Chlorophyll-*a* and Turbidity Sensors Technology

The first attempt to measure *in-situ* chlorophyll-*a* dates back to 1966 [59], and, since then, several efforts have been made to obtain reliable field type fluorometers. Actually, they can be subdivided into pumped flow-through models and not pumped ones: the first category comprises all sensors in which excitation and detection are internal to the instrument, whereas all sensors for which excitation and detection occur externally belong to the second class.

Commercial *in-situ* fluorometers use blue excitation light (typically LED 455/470 nm). This light is absorbed by phytoplankton pigments. After rapid photobiological processes in the cell, part of the absorbed energy is re-emitted as fluorescence by chlorophyll-*a* at 680 nm (centre of peak).

Many sensors combine fluorometer and turbidimeter since the scattered light can be used to measure both chlorophyll-*a* and turbidity using different wavelengths of about 470 nm and 700 nm, respectively.

Turbidity is properly used to describe water clarity since it provides an estimate of the attenuation of the light due to the presence of suspended materials measuring their effect on the optical properties of the sea water [60].

Despite the simplicity of the method and the relatively low complexity of the instrumentation, long term monitoring of chlorophyll-*a* is still a challenge due to fouling problems and to the variability of the observations. Indeed, being related to biological processes, the observations can be affected by phytoplankton composition and physiological status, morphology and other environmental factors such as presence of other plant pigments and of dissolved organic matter [61,62]. Indeed, suspended particles affect also turbidity measurements since their different size and distribution can influence the optical observations making it difficult to obtain reliable long term time series of turbidity measurements [37].

The intrinsic variability of the parameters to be monitored underlines the need to perform accurate calibration of the sensors to be deployed at sea in operational monitoring sites, in order to be able to verify drift of the sensor and to correct the data between two subsequent installations.

The most accurate method to analyze chlorophyll-*a* concentration in laboratory is HPLC [25], while other options are spectrophotometry and fluorometry [63].

*In situ* turbidimeters are mostly calibrated through standard reference such as formazine although this chemical provides a relative measurement that can vary not only between different types of sensors, but also from instrument to instrument, and, for this reason, an intercomparison in the same calibration bath of multiple sensors is highly recommended.

## References

[1] Lampitt R., Favali P., Barnes C.R., Church M.J., Cronin M.F., Hill K.L., Kaneda Y., Karl D.M., Knap A.H., McPhaden M.J. *In situ* Sustained Eulerian Observatories. Proceedings of the OceanObs’09: Sustained Ocean Observations and Information for Society.

[2] Garzoli S., Boebel O., Bryden H., Fine R., Fukasawa M., Gladyshev S., Johnson G., Johnson M., MacDonald A., Meinen C. Progressing Towards Global Sustained Deep Ocean Observations. Proceedings of OceanObs’09: Sustained Ocean Observations and Information for Society.

[3] Heimbürger L.E., Lavigne H., Migon C., D’Ortenzio F., Estournel C., Coppola L., Miquel J.C. (2013). Temporal variability of vertical export flux at the DYFAMED time-series station (Northwestern Mediterranean Sea). Prog. Oceanogr..

[4] Dickey T., Zedler S., Yu X., Doney S.C., Frye D., Jannasch H., Manov D., Sigurdson D., McNeil J.D., Dobeck L. (2001). Physical and biogeochemical variability from hours to years at the Bermuda Testbed Mooring site: June 1994–March 1998. Deep Sea Res. II Top. Stud. Oceanogr..

[5] García-Reyes M., Largier J.L., Sydeman W.J. (2014). Synoptic-scale upwelling indices and predictions of phyto- and zooplankton populations. Prog. Oceanogr..

[6] Hoteit I., Triantafyllou G., Petihakis G. (2004). Towards a data assimilation system for the Cretan Sea ecosystem using a simplified Kalman filter. J. Mar. Syst..

[7] Triantafyllou G., Petihakis G., Allen I.J. (2003). Assessing the performance of the Cretan Sea ecosystem model with the use of high frequency M3A buoy data set. Ann. Geophys..

[8] Marty J.C., Chiavérini J. (2002). Seasonal and interannual variations in phytoplankton production at DYFAMED time-series station, northwestern Mediterranean Sea. Deep Sea Res. II Top. Stud. Oceanogr..

[9] Faugeras B., Lévy M., Mémery L., Verron J., Blum J., Charpentier I. (2003). Can biogeochemical fluxes be recovered from nitrate and chlorophyll data? A case study assimilating data in the Northwestern Mediterranean Sea at the JGOFS-DYFAMED station. J. Mar. Syst..

[10] De Fommervault O.P., Migon C., D’Ortenzio F., d’Alcalà M.R., Coppola L. (2015). Temporal variability of nutrient concentrations in the northwestern Mediterranean sea (DYFAMED time-series station). Deep Sea Res. I Oceanogr. Res. Pap..

[11] Drakopoulos P., Petihakis G., Valavanis V., Nittis K., Triantafyllou G. (2003). Optical variability associated with phytoplankton dynamics in the Cretan Sea during 2000 and 2001. Building the European Capacity in Operational Oceanography.

[12] Cianca A., Santana R., Hartman S.E., Martín-González J.M., González-Dávila M., Rueda M.J., Llinás O., Neuer S. (2013). Oxygen dynamics in the North Atlantic subtropical gyre. Deep Sea Res. II Top. Stud. Oceanogr..

[13] Lindstrom E., Gunn J., Fischer A., McCurdy A., Glover L.K., Members T.T. (2012). A Framework for Ocean Observing.

[14] National Research Council (2003). Enabling Ocean Research in the 21st Century: Implementation of a Network of Ocean Observatories.

[15] Cronin M.F., Weller R.A., Lampitt R.S., Send U., Rustamov R.B., Salahova S.E. (2002). Ocean reference stations. Earth Observation.

[16] Huang Y., Schmitt F.G. (2014). Time dependent intrinsic correlation analysis of temperature and dissolved oxygen time series using empirical mode decomposition. J. Mar. Syst..

[17] Kuss J., Roeder W., Wlost K.P., DeGrandpre M.D. (2006). Time-series of surface water CO_2_ and oxygen measurements on a platform in the central Arkona Sea (Baltic Sea): Seasonality of uptake and release. Mar. Chem..

[18] Martini S., Nerini D., Tamburini C. (2014). Relation between deep bioluminescence and oceanographic variables: A statistical analysis using time-frequency decompositions. Prog. Oceanogr..

[19] Bergondo D.L., Kester D.R., Stoffel H.E., Woods W.L. (2005). Time-series observations during the low sub-surface oxygen events in Narragansett Bay during summer 2001. Mar. Chem..

[20] Waniek J.J., Schulz-Bull D.E., Kuss J., Blanz T. (2005). Long time series of deep water particle flux in three biogeochemical provinces of the northeast Atlantic. J. Mar. Syst..

[21] Martini M., Butman B., Mickelson M. (2007). Long-Term Performance of AanderaaOptodes and Sea-Bird SBE-43 Dissolved-Oxygen Sensors Bottom Mounted at 32 m in Massachusetts Bay. J. Atmos. Ocean. Technol..

[22] Bittig H.C., Fiedler B., Steinhoff T., Körtzinger A. (2012). A novel electrochemical calibration setup for oxygen sensors and its use for the stability assessment of Aanderaa optodes. Limnol. Oceanogr. Meth..

[23] Gruber N., Doney S.C., Emerson S.R., Gilbert D., Kobayashi T., Körtzinger A., Johnson G.C., Johnson K.S., Riser S.C., Ulloa O. Adding oxygen to argo: Developing a global *in situ* observatory for ocean deoxygenation and biogeochemistry. Proceedings of the OceanObs’09: Sustained Ocean Observations and Information for Society.

[24] Takeshita Y., Martz T.R., Johnson K.S., Plant J.N., Gilbert D., Riser S.C., Neill C., Tilbrook B. (2013). A climatology-based quality control procedure for profiling float oxygen data. J. Geophys. Res. Oceans.

[25] Murray A.P., Gibbs C.F., Longmore A.R., Flett D.J. (1986). Determination of chlorophyll in marine waters: Intercomparison of a rapid HPLC method with full HPLC, spectrophotometric and fluorometric methods. Mar. Chem..

[26] Strickland J.D.H., Parsons T.R. (1972). A Practical Handbook of Seawater Analysis.

[27] Dickson A.G. (1996). Determination of dissolved oxygen in seawater by Winkler titration. WOCE Operations Manual.

[28] Carpenter J.H. (1965). The Chesapeake Bay Institute technique for the Winkler dissolved oxygen method. Limnol. Oceanogr..

[29] Carpenter J.H. (1965). The accuracy of the Winkler method for dissolved oxygen. Limnol. Oceanogr..

[30] Carpenter J.H. (1966). New measurements of oxygen solubility in pure and natural waters. Limnol. Oceanogr..

[31] Murray C.N., Riley J.P. (1969). The solubility of gases in distilled water and sea water—II. Oxygen. Deep-Sea Res. Oceanogr. Abstr..

[32] Coppola L., Salvetat F., Delauney L., Machoczek D., Karstensen J., Sparnocchia S., Thierry V., Hydes D., Haller M., Nair R. (2013). White Paper on Dissolved Oxygen Measurements: Scientific Needs and Sensors Accuracy.

[33] Owens W.B., Millard R.C. (1985). A New Algorithm for CTD Oxygen Calibration. J. Phys. Oceanogr..

[34] Joos F., Plattner G.K., Stocker T.F., Körtzinger A., Wallace D.W.R. (2003). Trends in marine dissolved oxygen: Implications for ocean circulation changes and the carbon budget. EOS Trans. Am. Geophys. Union.

[35] Bittig H.C., Fiedler B., Scholz R., Krahmann G., Körtzinger A. (2014). Time response of oxygen optodes on profiling platforms and its dependence on flow speed and temperature. Limnol. Oceanogr. Meth..

[36] Hongve D., Kesson G. (1998). Comparison of nephelometric turbidity measurements using wavelengths 400–600 and 860 nm. Water Res..

[37] Downing J. (2004). Turbidity Monitoring. Environmental Instrumentation and Analysis Handbook.

[38] Carrol M., Chigounis D., Gilbert S., Gundersen K., Hayashi K., Janzen C., Johengen T., Koles T., Laurier F., McKissack T. (2006). Performance Verification Statement for the Wet Labs ECO FLNTUSB Fluorometer.

[39] Van Heukelem L., Thomas C.S. (2001). Computer-assisted high-performance liquid chromatography method development with applications to the isolation and analysis of phytoplankton pigments. J. Chromatogr. A.

[40] D’Asaro E.A., McNeil C. (2013). Calibration and Stability of Oxygen Sensors on Autonomous Floats. J. Atmos. Ocean. Technol..

[41] Wanninkhof R., Asher W.E., Ho D.T., Sweeney C., McGillis W.R. (2009). Advances in Quantifying Air-Sea Gas Exchange and Environmental Forcing. Annu. Rev. Mar. Sci..

[42] Cullen J.J., Davis F. (2003). The blank can make a big difference in oceanographic measurements. Limnol. Oceanogr. Bull..

[43] Goodin D.G., Han L., Fraser R.N., Rundquist D.C., Stebbins W.A. (1993). Analysis of suspended solids in water using remotely sensed high resolution derivative spectra. Photogramm. Eng. Remote Sens..

[44] Twardowski M.S., Claustre H., Freeman S.A., Stramski D., Huot Y. (2007). Optical backscattering properties of the “clearest” natural waters. Biogeosciences.

[45] Earp A., Hanson C.E., Ralph P.J., Brando V.E., Allen S., Baird M., Clementson L., Daniel P., Dekker A.G., Fearns P.R.C.S. (2011). Review of fluorescent standards for calibration of *in situ* fluorometers: Recommendations applied in coastal and ocean observing programs. Opt. Express.

[46] Diehl H., Markuszewski R. (1989). Studies on fluorescein—VII: The fluorescence of fluorescein as a function of pH. Talanta.

[47] Sjöback R., Nygren J., Kubista M. (1995). Absorption and fluorescence properties of fluorescein. Spectrochim. Acta A Mol. Biomol. Spectrosc..

[48] Esteves V.I., Santos E.B.H., Duarte A.C. (1999). Study of the effect of pH, salinity and DOC on fluorescence of synthetic mixtures of freshwater and marine salts. J. Environ. Monit..

[49] Bozzano R., Pensieri S., Pensieri L., Cardin V., Brunetti F., Bensi M., Petihakis G., Tsagaraki T.M., Ntoumas M., Podaras D. The M3A Network of Open Ocean Observatories in the Mediterranean Sea. Proceedings of the OCEANS 2013 MTS/IEEE.

[50] Lampitt R., Cristina L. FixO3 Network Project: Integration, Harmonization and Innovation. Proceedings of the European Geosciences Union General Assembly 2016.

[51] Canepa E., Pensieri S., Bozzano R., Faimali M., Traverso P., Cavaleri L. (2015). The ODAS Italia 1 buoy: More than forty years of activity in the Ligurian Sea. Prog. Oceanogr..

[52] Edwards B., Murphy D., Janzen C., Larson N. (2010). Calibration, Response, and Hysteresis in Deep-Sea Dissolved Oxygen Measurements. J. Atmos. Ocean. Technol..

[53] Carlson J. (2010). Development of an optimized dissolved oxygen sensor for oceanographic profiling. Int. Ocean Syst..

[54] Clark L.C., Wolf R., Granger D., Taylor Z. (1953). Continuous recording of blood oxygen tensions by polarography. J. Appl. Physiol..

[55] Garcia H.E., Gordon L.I. (1992). Oxygen solubility in sea water: Better fitting equations. Limnol. Oceanogr..

[56] Demas J.N., de Graff B.A., Coleman P.B. (1999). Oxygen sensors based on luminescence quenching. Anal. Chem..

[57] Klimant I., Kühl M., Glud R.N., Holst G. (1997). Optical measurement of oxygen and temperature in microscale: Strategies and biological applications. Sens. Actuators B Chem..

[58] Tengberg A., Hovdenes J., Andersson H.J., Brocandel O., Diaz R., Hebert D., Arnerich T., Huber C., Körtzinger A., Khripounoff A. (2006). Evaluation of a lifetime-based optode to measure oxygen in aquatic systems. Limnol. Oceanogr. Meth..

[59] Lorenzen C.J. (1966). A method for the continuous measurement of *in vivo* chlorophyll concentration. Deep Sea Res. Oceanogr. Abstr..

[60] Boss E., Taylor L., Gilbert S., Gundersen K., Hawley N., Janzen C., Johengen T., Purcell H., Robertson C., Schar D.W.H. (2009). Comparison of inherent optical properties as a surrogate for particulate matter concentration in coastal waters. Limnol. Oceanogr. Meth..

[61] Falkowski P., Kiefer D.A. (1985). Chlorophyll-*a* fluorescence in phytoplankton: Relationship to photosynthesis and biomass. J. Plankton Res..

[62] Suggett D.J., MacIntyre H.L., Geider R.J. (2004). Evaluation of biophysical and optical determinations of light absorption by photosystem II in phytoplankton. Limnol. Oceanogr. Meth..

[63] Suggett D.J., Prasil O., Borowitzka M.A. (2011). Chlorophyll a Fluorescence in Aquatic Sciences: Methods and Applications.

